# Mechanical Properties of 3D-Printed ABS Composites Reinforced with Multi-Scale Carbon/Kevlar Hybrid Fibers

**DOI:** 10.3390/ma19132690

**Published:** 2026-06-23

**Authors:** Shaoqi Dong, Shixian Li, Wanying Zhu

**Affiliations:** 1 Key Laboratory of Traffic Safety on Track of Ministry of Education, School of Traffic & Transportation Engineering, Central South University, Changsha 410075, China; 234211055@csu.edu.cn; 2 ICUBE Laboratory—CNRS, University of Strasbourg, 67000 Strasbourg, France

**Keywords:** multi-scale hybrid fiber reinforcements, continuous carbon fiber, continuous Kevlar fiber, flexural behavior, interlayer behavior, hybrid effect, 3D printing

## Abstract

**Highlights:**

**What are the main findings?**

- Multi-scale carbon/Kevlar hybrids were fabricated in FDM-printed ABS composites.
- Short-fiber-filled matrices improved flexural properties and peel resistance.
- CCF + ABS/SCF achieved the highest flexural modulus and strength, CKF + ABS/SCF offers balanced load-bearing and deformation capability.

**What are the implications of the main findings?**

- Multi-scale hybridization tailors flexural and interlaminar performance.
- Fiber matching guides high-stiffness or balanced structural design.
- Hybrid composites showed positive deviations from the RoM reference.

**Abstract:**

Fused deposition modeling (FDM) provides a flexible manufacturing route for continuous fiber-reinforced thermoplastic composites, but weak interlaminar bonding and the trade-off between load-bearing capacity and deformation capability still limit their structural applications. In this study, multi-scale carbon/Kevlar fiber hybridization was introduced into acrylonitrile butadiene styrene (ABS)-based composites by combining continuous carbon fiber (CCF) or continuous Kevlar fiber (CKF) with short carbon fiber-filled ABS (ABS/SCF) or short Kevlar fiber-filled ABS (ABS/SKF). Four hybrid configurations and two continuous-fiber baseline composites were fabricated by FDM and evaluated through three-point bending tests, floating roller peel tests, peeled-surface SEM observations, and Rule-of-Mixtures-based hybrid effect analysis. The flexural results showed that short-fiber-filled matrices improved the flexural properties of both CCF- and CKF-based composites, but the degree of improvement depended on the fiber combination. Among the investigated configurations, CCF + ABS/SCF exhibited the highest flexural modulus and strength, which were 34.31% and 27.26% higher than those of CCF + ABS, respectively. For the CKF-based composites, CKF + ABS/SCF increased the flexural modulus and strength by 31.51% and 26.78%, compared with CKF + ABS, while maintaining the progressive deformation behavior associated with Kevlar reinforcement. The peel results showed that all hybrid composites had higher interlaminar peel resistance than their corresponding baselines, with increases ranging from 18.66% to 54.42%. The peeled-surface SEM observations indicated that the short-fiber-filled matrices changed the crack-propagation features, with more matrix tearing, fiber pull-out, and irregular peeling areas. The RoM-based comparison showed that the measured flexural properties of all hybrid configurations were higher than the corresponding RoM reference values. Overall, CCF + ABS/SCF was more suitable for improving stiffness and load-bearing capacity, whereas CKF + ABS/SCF showed a more balanced response in terms of flexural performance, interlaminar peel resistance, and progressive deformation behavior.

## 1. Introduction

The demand for fiber-reinforced polymer composites is steadily increasing in the transportation, energy equipment, and other lightweight structure sectors [1,2,3,4,5]. This is due to the high specific strength, high specific stiffness, and designable mechanical properties of fiber-reinforced composites [6,7]. Traditional composite manufacturing techniques can produce high-performance components. However, they usually rely on molds, have relatively long processing cycles, and have limitations in manufacturing complex and customized structures [8,9]. Compared to these traditional manufacturing methods, additive manufacturing (AM), also known as 3D printing, provides a more flexible and cost-effective method for producing composite components with complex geometries and customized structures [10,11]. With the continuous improvement of printable materials and processing strategies, 3D-printed composite components have not only become conceptual products but are increasingly being used as functional structural components [12].

Among various additive manufacturing technologies, fused deposition modeling (FDM) is one of the most widely used methods for manufacturing thermoplastic composites because of its simple process and broad material applicability [12,13,14]. However, the polymer composites printed by FDM are still limited by material and process-related defects [15,16]. During the layer-by-layer deposition process, the absence of external consolidation pressure and insufficient molecular diffusion between adjacent deposited roads can lead to incomplete interlayer bonding, internal voids, and weak interlayer interactions [17]. These defects reduce the load transfer efficiency and make the printed components prone to delamination and premature failure when subjected to mechanical loads [17,18,19].

For FDM-printed thermoplastic composites, it remains challenging for a single continuous fiber-reinforced system to simultaneously achieve a comprehensive improvement in high flexural stiffness, high strength, and stable damage tolerance [20]. One effective way to overcome this limitation is through hybrid fiber reinforcement, which is achieved by combining different scales of reinforcing materials or different types of fibers within the same printing structure. Qu et al. [21] proposed a continuous carbon fiber/obsidian fiber hybrid reinforcement dual-matrix extrusion printing method. Distributing more obsidian fibers in the base layer can effectively enhance the bending energy absorption capacity, with the maximum improvement reaching 126.1%. They also proposed a method for optimizing the structural design. Zhao et al. [22] proposed a functional gradient hybrid strategy, achieving gradient changes in fiber content and material properties between layers and within layers, which enabled the hybrid fiber-reinforced structure to increase the flexural modulus and interlayer shear strength by 25.95% and 41.20% respectively, effectively suppressing the occurrence of delamination damage. These studies demonstrate that compared to a single-scale reinforcing system, hybrid fiber reinforcement can effectively enhance the stiffness and strength of the material. However, existing research often focuses more on interlayer fiber hybrid reinforcement systems, mainly concentrating on optimizing the layup settings and fiber content. Huang and Joosten [23] designed and characterized 3D printed continuous fiber hybrid laminates composed of polyamide/glass fiber and polyamide/carbon fiber. Through changing the thickness of the carbon fiber layer and the ratio of the carbon fiber/glass fiber layers, the study explored the pseudo-elastic response of 3D printed thermoplastic hybrid composites, demonstrating that the combination of different types of continuous fibers can improve the problem of difficult balance between stiffness and deformation capacity in the material. Research on intralayer hybrid fibers is relatively scarce, especially the multi-scale hybridization of different performance fibers within the layer has received little attention. Multi-scale hybridization enhancement is a highly promising strategy. By hybridizing two materials within the layer, it may have an impact on the comprehensive mechanical properties and interlayer interface bonding of the composite. Therefore, it is necessary to conduct research on this.

To address these issues, this study investigated the bending and interlaminar behaviors of 3D-printed ABS-based composites reinforced by multi-scale carbon fiber/Kevlar fiber mixtures. Four hybrid structures were designed by combining continuous carbon fiber (CCF) or continuous Kevlar fiber (CKF) with short carbon fiber-filled ABS (ABS/SCF) or short Kevlar fiber-filled ABS (ABS/SKF). Two continuous fiber composites were used as reference materials. Three-point bending tests were conducted to evaluate the flexural modulus, flexural strength, and energy absorption behavior of the composites, since flexural performance is an important consideration for printed composite components used in lightweight load-bearing structures. Under flexural loading, the printed composites experience combined tensile, compressive, and interlaminar shear responses, which allows the contributions of the continuous fibers, short-fiber-filled ABS matrix, and printed interlayer interfaces to be evaluated in a unified loading condition. Floating roller peel tests were performed to assess the interlaminar peel resistance, and the representative peeled surface morphology was examined to clarify the crack propagation characteristics. Additionally, a RoM-based comparison was used to evaluate the hybrid effect by calculating the deviation of the measured flexural properties from the corresponding RoM reference values. It is expected that these results will provide guidance for selecting hybrid structures based on different design goals, such as high flexural stiffness, enhanced peel strength, or balanced stiffness-ductility performance.

## 2. Materials and Methods

### 2.1. Raw Materials

In this study, three types of thermoplastic matrix filaments were used as matrix materials, including pure acrylonitrile-butadiene-styrene (ABS) material, short carbon fiber-filled ABS (ABS/SCF) material, and short Kevlar fiber-filled ABS (ABS/SKF) material. These filaments were provided by NANOVIA (ZA de Saint Paul, Louargat, France). Continuous carbon fiber (CCF, T300B, 1K, Toho Tenax Co., Ltd., Tokyo, Japan) and continuous Kevlar fiber (CKF, Kevlar 29, 600D, DuPont, Wilmington, DE, USA) were used as continuous reinforcing phases. These two types of continuous fibers were selected to maintain a comparable level of continuous reinforcement in different composite structures, so as to be able to evaluate the effects of fiber type and hybrid structure under similar processing conditions. The material specifications are shown in Table 1. All the materials were stored in sealed containers with desiccants, and were dried at 60 °C for 4 h before printing to reduce the impact of water absorption on extrusion stability, interlayer bonding, and mechanical properties.

### 2.2. Printing Parameters and Specimen Fabrication

Six composite configurations were designed to investigate the effects of continuous fiber type and short-fiber-filled matrix type on the flexural and interlaminar properties of FDM-printed ABS composites. Among them, two continuous fiber composites: CCF + ABS and CKF + ABS, were used as the reference composites. In these two materials, pure ABS matrix was combined with CCFs or CKF. Based on these two reference composites, four multi-scale hybrid composites were further prepared by replacing pure ABS with short fiber-filled ABS matrix, including CCF + ABS/SCF, CCF + ABS/SKF, CKF + ABS/SCF, and CKF + ABS/SKF. According to the interrelationship between the continuous fibers and the short fibers in the matrix, these four hybrid composites were classified as homogeneous hybrid composites and heterogeneous hybrid composites. The above composite configurations and their classification are summarized in Table 2, which enables researchers to directly compare the performance of these two different hybrid modes.

**Figure 1:**
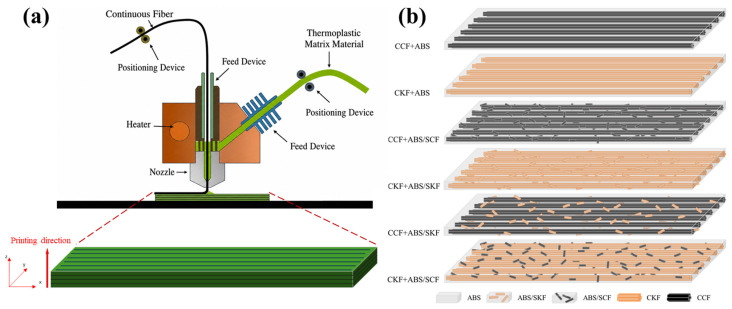
Fabrication process and laminate architectures of the printed composites: (**a**) continuous-fiber FDM process; (**b**) laminate configurations.

In this design scheme, continuous fibers were introduced as the main reinforcing phase, while the short-fiber-filled matrix was used to improve the regions dominated by the matrix in the printed composites. To ensure that the different composite configurations could be compared under similar structural conditions, all specimens were designed with the same symmetric layer arrangement and printing strategy. All specimens were prepared using the COMBOT-200 desktop fused deposition modeling (FDM) printer (Shenzhen Xietong Innovation High-Tech Development Co., Ltd., Shenzhen, China). To eliminate the influence of process fluctuations, all composite configurations adopted the same printing strategy and process parameters. The printing principle and composition diagram are shown in Figure 1a, and the sample material diagram is shown in Figure 1b.

During the printing process, the nozzle temperature, platform temperature, and printing speed were set at 250 °C, 100 °C, and 3 mm/s, respectively. The temperature was selected according to the recommended printing temperature range provided by the material supplier to ensure sufficient flowability of the ABS-based matrix during extrusion [24]. The layer height was fixed at 0.4 mm, the laying line width was set at 1.4 mm, and the extrusion ratio was maintained at 1.10. A relatively low printing speed and controlled extrusion ratio were used to increase the contact time between the molten matrix and the continuous fibers, thereby promoting stable fiber/matrix contact during deposition [6]. The continuous fibers were deposited along a unidirectional path parallel to the longitudinal axis (*X*-axis) of the specimens, yielding a fiber layup angle of 0°. All specimens were directly printed according to the required geometric configurations for the corresponding mechanical tests. Specifically, the three-point bending samples were fabricated according to ISO 14125 [28]; they featured a nominal thickness of 4.0 mm and comprised 10 layers. The floating roller peel samples were prepared based on the rigid and flexible section dimensions described in ASTM D3167 [29], where the rigid section (1.6 mm thick) and flexible section (0.8 mm thick) were composed of 4 layers and 2 layers, respectively. After completion, any samples with obvious printing defects or severe size deviations were scrapped.

### 2.3. Mechanical Testing and Microstructural Characterization

**Figure 2:**
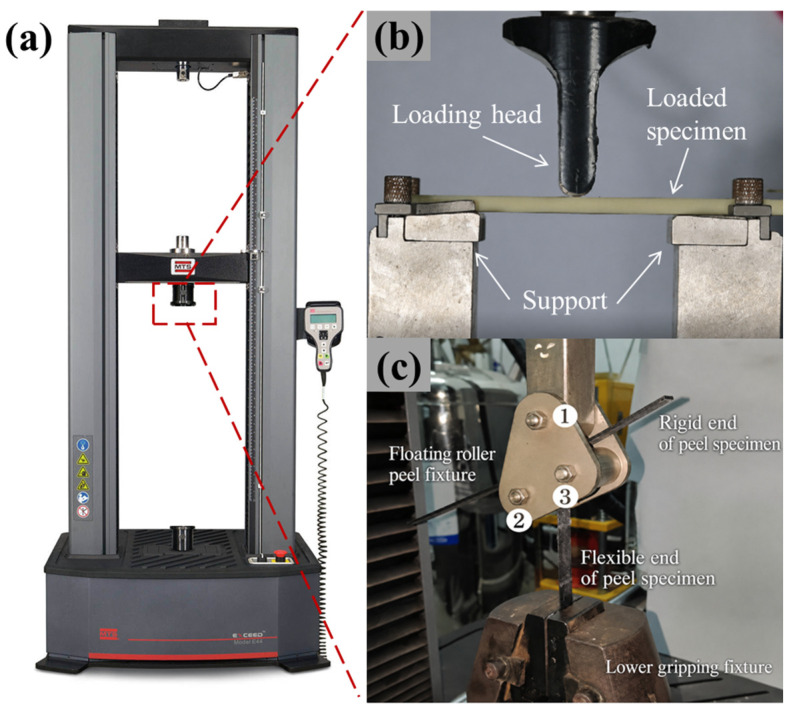
Mechanical testing platform: (**a**) Universal testing machine; (**b**) Three-point bending test; (**c**) Floating roller peel test.

All mechanical tests were conducted at room temperature and performed using an MTS Exceed E44 (MTS Systems Corporation, Eden Prairie, MN, USA) testing machine equipped with high-precision force sensors. For each composite configuration, 5 replicate samples were tested and the average values were compared. Firstly, the flexural performance of the composites was evaluated through the three-point bending test, and then the interlaminar bonding and delamination resistance were assessed through the floating roller peel test. The schematic diagram of the experimental equipment is shown in Figure 2.

The three-point bending test was conducted according to the ISO 14125 standard to evaluate the flexural performance of the printed composites [28]. The samples were directly printed with a length of 80 mm, a width of 7 mm, and a thickness of 4 mm. The test was carried out at a transverse speed of 2.0 mm/min. During the test, the load–displacement data was continuously recorded and converted into a bending stress–strain curve. The flexural modulus of the material was determined from the linear strain range of 0.05% to 0.25%. The flexural modulus (*E*_*f*_) was calculated using Equation (1):

(1) $$E_{f}=\frac{L^{3}F}{4bH^{3}\delta_{f}}$$

where *δ_f_* is the deflection of the specimen under the corresponding load, *L* is the test span (mm), *F* is the applied load (kN), *b* is the width of the specimen (mm), *H* is the thickness of the specimen (mm).

The flexural strength (*σ*_*f*_) was calculated from the maximum stress using Equation (2):

(2) $$\sigma_{f}=\frac{3LP_{max}}{2bH^{2}}$$

where *P*_max_ is the maximum load recorded during the flexural test (kN), *L* represents the test span (mm), *b* represents the width of the specimen (mm), *H* represents the thickness of the specimen (mm).

For each composite configuration, five replicate samples were tested, and the reported results were presented as the average value and standard deviation. The area under the flexural stress–strain curve was used to compare the energy-absorption behavior during damage development.

The floating roller peel test was conducted according to the ASTM D3167 standard to evaluate the interlaminar bonding and delamination resistance of the printed composites [29]. Each peel sample consisted of a rigid upper part and a flexible lower part. The thickness of the rigid part was 1.6 mm, and the length was 161.2 mm, while the thickness of the flexible part was 0.8 mm, and the length was 200 mm. The width of all peel samples was fixed at 12.6 mm. During sample preparation, a pre-crack of 25.4 mm in length was introduced at one end of the interface to promote crack formation. The peel speed was set at 100 mm/min. By analyzing the stable crack propagation area in the load–displacement curve, the average peel load was determined. The formula for calculating peel strength (*σ_p_*) is as shown in Equation (3):

(3) $$\sigma_{p}=\frac{F_{\mathrm{mean}}}{b}$$

where *F*_mean_ represents the average peel force (N), and *b* is the width of the specimen (m).

To evaluate the statistical significance of the interlaminar peel property variations among the investigated composite configurations, a one-way analysis of variance (ANOVA) was performed specifically on the peel test data, followed by Tukey’s honest significant difference (HSD) post hoc test. The significance level was set at *α* = 0.05. All statistical calculations were executed based on the sample size of five replicates per configuration (*n* = 5), with results expressed as mean ± standard deviation.

After the floating roller peel test, the selected representative samples were observed using a field emission scanning electron microscope (FESEM, Hitachi S-4800, Hitachi High-Technologies Corporation, Tokyo, Japan). The focus of the observation was on interface debonding, fiber pull-out, local matrix tearing, and crack path deviation.

### 2.4. Evaluation of Hybrid Effect

To evaluate the hybrid effect of the investigated composites, a volume-fraction-based Rule of Mixtures (RoM) approach was used as a reference for comparison. In this study, the hybrid effect was defined as the normalized deviation of an experimentally measured mechanical property from the corresponding RoM reference value. It was used as a reference system for comparing different hybrid configurations. The theoretical property of a hybrid composite, PROM was calculated as shown in Equation (4):

(4) $$P_{\mathrm{ROM}}=\lambda_{c}P_{c}+\lambda_{s}P_{s}$$

where *P_c_* and *P_s_* represent the reference properties associated with the continuous-fiber component and the short fiber-reinforced matrix component, respectively. And *λ_c_* and *λ_s_* are the corresponding normalized volume fractions, with *λ_c_ + λ_s_* = 1.

The hybrid effect coefficient, *HE* was then defined as Equation (5):

(5) $$HE=\frac{P_{\exp}-P_{\mathrm{ROM}}}{P_{\mathrm{ROM}}}$$

where *P_exp_* is the experimentally measured property of the hybrid composite. A positive *HE* value indicates that the measured property is higher than the corresponding RoM reference value, whereas a negative value indicates that the measured property is lower than the reference value. It should be noted that the HE value quantifies the deviation of the experimental result from the linear reference value, but does not by itself identify the specific microscopic mechanism responsible for the deviation.

The volume-fraction parameters used for the RoM-based calculation are summarized in Table 3. These parameters were derived from the printing track geometry and the estimated fiber volume fractions, and were used consistently for all hybrid configurations. In this study, the hybrid effect was calculated specifically for flexural strength and flexural modulus to compare the synergistic efficiency of different hybrid configurations.

## 3. Results and Discussion

### 3.1. Flexural Response and Mechanical Reinforcement of Hybrid Composites

#### 3.1.1. Flexural Stress–Strain Response

**Figure 3:**
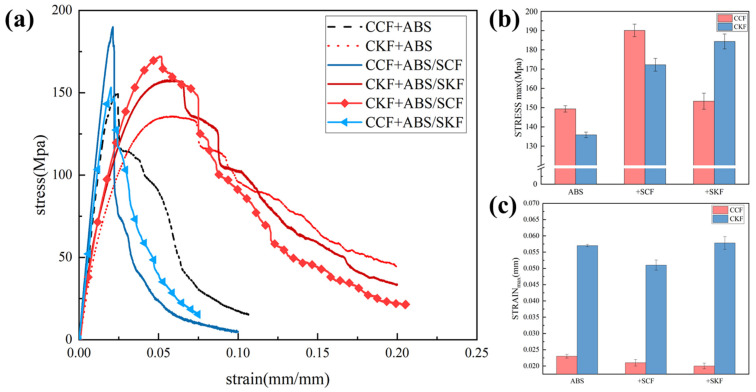
Three-point bending behavior of 3D-printed fiber-reinforced ABS composites: (**a**) representative stress–strain curves; (**b**) peak stress; and (**c**) strain corresponding to peak stress.

Figure 3 shows the bending stress–strain curve of the 3D-printed ABS-based composite and its corresponding peak bending parameters. In Figure 3a, all specimens exhibit approximately linear responses in the initial loading stage, indicating that the applied load increases proportionally with deformation and no significant macroscopic damage occurs within the small strain range. As the bending strain increases, the curve gradually deviates from the initial linear region, entering the nonlinear damage propagation stage, and then the material fails. The curve indicates that the flexural performance of all materials can be roughly divided into two types. One is the material containing CCF, which can withstand higher stress under smaller deformation, reaching the peak stress at a relatively lower strain level, and then the stress drops rapidly, resulting in a more sudden failure process. The other is the material containing continuous Kevlar, which can withstand lower maximum stress but has a wider strain range, showing a more gradual failure form, and presenting a relatively smoother failure process. These phenomena indicate that the addition of short fibers does not significantly change the failure mode of the material, and the failure form of the hybrid fiber composite is still dominated by the continuous fibers. Although the overall failure mode was still mainly governed by the continuous fiber type, the variations in the three-point bending curves and peak parameters shown in Figure 3b and c indicate that short-fiber filling affected the stiffness, strength, and local damage characteristics of the composites. For example, CKF + ABS/SCF showed a more rigid response during failure.

To specifically compare the changes in the values of flexural modulus and flexural strength, the data are summarized in Table 4. For the composites containing CCF, after introducing short carbon fibers, the flexural modulus of CCF + ABS/SCF increased to 10,532.51 ± 579.29 MPa, and the flexural strength reached 190.06 ± 10.07 MPa, with the increases reaching 34.31% and 27.26% respectively. In contrast, CCF + ABS/SKF showed only limited improvement, with their flexural modulus and strength increasing by 7.43% and 2.66% respectively. This result indicates that filling short carbon fibers can effectively enhance the bending stiffness and bearing capacity of CCF composites. The effect of chopped Kevlar fibers in improving the bending resistance is relatively weak. For the composites containing Kevlar fibers, the filling of both short fibers has improved the flexural performance of the composite based on CKF. Among them, CKF + ABS/SCF achieved a flexural modulus of 7540.29 ± 505.20 MPa and a flexural strength of 172.23 ± 10.68 MPa, corresponding to 31.51% and 26.78% increases respectively. CKF + ABS/SKF also showed an improvement in flexural performance, with its modulus increasing by 32.09% and its strength increasing by 16.29%. These results indicate that filling short fibers in the matrix can partially compensate for the relatively lower bending stiffness of the CKF system. The different reinforcement efficiencies can be attributed to the stiffness difference between SCF and SKF, the matrix stiffening effect, and the matching relationship between the continuous fiber and the short-fiber-filled matrix. Previous studies on FDM-printed short-carbon-fiber-reinforced ABS have shown that the incorporation of rigid short carbon fibers can improve the stiffness and load-bearing capacity of printed parts by strengthening the matrix-dominated regions and promoting local stress transfer [30]. This explains why ABS/SCF produced more obvious improvements than ABS/SKF in both the CCF and CKF-based composites. In the present study, the CCF + ABS/SCF exhibited the highest flexural modulus and strength, indicating that the carbon/carbon multi-scale hybrid design is more suitable for stiffness- and strength-dominated applications. Similar stiffness-dominated reinforcement effects of continuous carbon fibers have also been reported in FDM-printed continuous-fiber-reinforced thermoplastic composites, although their final performance is strongly affected by fiber orientation, printing configuration, and interfacial bonding quality [25]. In comparison, SKFs are more likely to contribute to local deformation coordination and damage tolerance rather than direct stiffness enhancement [8,31]. Therefore, for the CCF-based composites, the specimens tended to fail rapidly after reaching the peak stress, and the toughening contribution of SKF could not be fully activated before final failure. For the CKF-based composites, however, the higher deformation capacity of CKF allowed the short-fiber-filled matrix to contribute more effectively to the overall flexural response, which explains the obvious improvement observed in both CKF + ABS/SCF and CKF + ABS/SKF.

**Figure 4:**
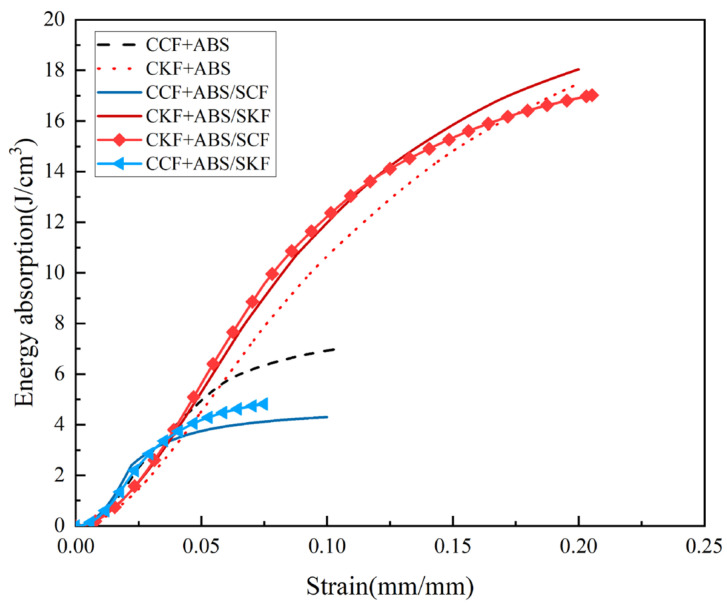
Energy absorption–strain curves of all composite configurations.

By integrating the area under the curve below the bending stress–strain graph, the corresponding energy absorption-strain curve was obtained, as shown in Figure 4. At relatively low strain levels, the hybrid composites typically exhibit higher cumulative energy absorption capacity compared to their corresponding continuous fiber benchmark composites, indicating that the short fiber-filled matrix improves the material’s response at the loading initiation stage. This improvement is particularly significant in materials containing SCF, suggesting that short carbon fibers are more effective in increasing the initial energy absorption rate than short Kevlar fibers. As the strain increases, the material’s energy absorption behavior becomes more dependent on the type of continuous fibers. For composites based on CCF, with their higher stress levels and stiffness, they can rapidly accumulate energy in the initial stage. However, due to the limited deformation capacity of the carbon fiber matrix, their energy absorption curves often end at a lower strain level. In contrast, the composites containing CKF exhibit a slower growth rate of energy absorption in the small strain stage, but can continuously accumulate energy over a wider range of strains. This phenomenon reflects the superior deformation ability of the CKF matrix and its more gradual damage expansion process. Among various materials, the combination of CKF + ABS/SCF shows an ideal balance: the short carbon fiber-filled matrix enhances the material’s stress level and initial energy absorption capacity, while the CKF help maintain the material’s deformation ability under larger strains. The energy absorption results clearly reveal the distinct contrast between these two types of continuous fiber composites: the composites based on CCF are characterized by rapid energy accumulation in the initial stage but limited strain limit; while the composites based on CKF exhibit a more sustained energy absorption capacity. The introduction of short carbon fibers is more effective in enhancing the early energy absorption capacity, and the damage-related energy-absorption response is mainly controlled by the deformation ability of the continuous fiber matrix. This conclusion is consistent with the previous analysis.

Across all configurations, although the modulus and strength of the CKF + ABS/SCF system did not reach the levels achieved by the CCF + ABS/SCF system, it nevertheless demonstrated significant improvements compared to the CKF + ABS system. The short carbon fibers proved more effective in compensating for the stiffness deficiencies inherent in the continuous Kevlar system. Concurrently, the system retained the more gradual deformation characteristics typical of CKF-reinforced materials, thereby offering distinct advantages in terms of energy absorption. Consequently, from a multidimensional perspective, CKF + ABS/SCF may be regarded as a more balanced configuration.

#### 3.1.2. Damage-Evolution Process Under Flexural Loading

**Figure 5:**
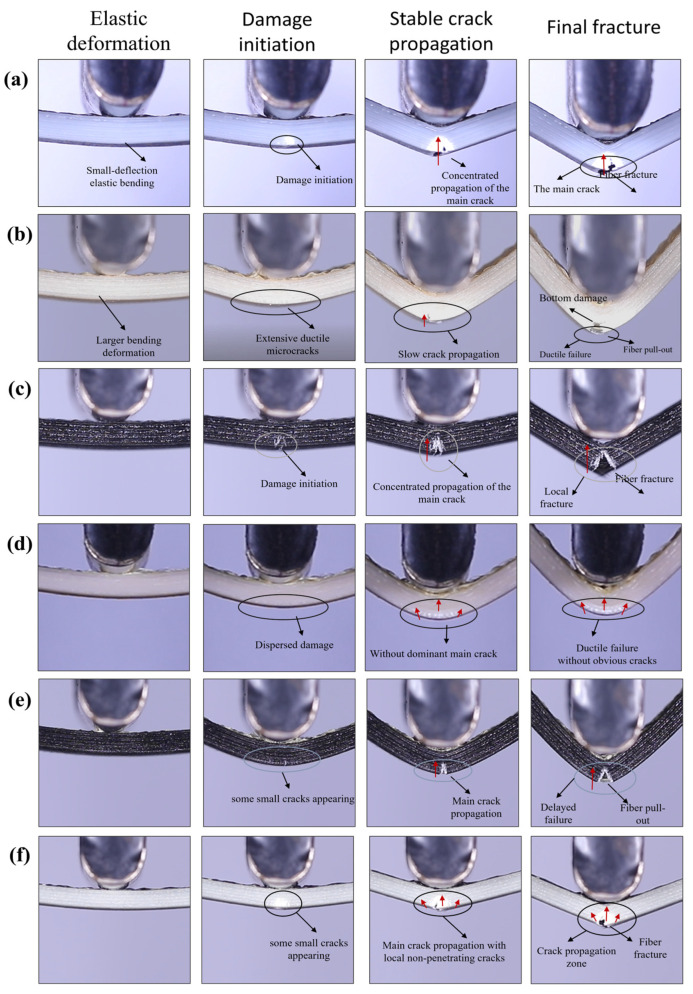
Damage-evolution process during three-point bending: (**a**) CCF + ABS; (**b**) CKF + ABS; (**c**) CCF + ABS/SCF; (**d**) CKF + ABS/SKF; (**e**) CKF + ABS/SCF; and (**f**) CCF + ABS/SKF. Red arrows indicate the main crack-propagation paths.

The deformation and damage evolution process of the representative sample during the three-point bending process is shown in Figure 5. These images can further link the bending curve with the macroscopic failure behavior. For composites, the samples usually undergo a similar damage sequence, including elastic deformation, damage initiation, crack propagation, and ultimate failure [32].

Comparisons reveal that the overall failure trend of all types of materials is controlled by the continuous fiber matrix. Materials containing CCF have relatively smaller deflections in the elastic stage. Subsequently, local damage occurs near the tension side of the mid-span region, followed by concentrated crack propagation along the thickness direction. This behavior is consistent with the steep stress–strain response and rapid stress drop after the peak discussed earlier, indicating that the failure mode is relatively brittle, mainly influenced by the high stiffness of carbon fibers and the low strain loss of carbon fibers. In contrast, CKF samples exhibit greater bending deformation during loading, with slower damage development, and the sample still maintains visible deformation until ultimate failure. CKF provides better deformation coordination and delays the sudden expansion of cracks. With the introduction of short fiber-filled matrix, the overall failure trend is still mainly controlled by the continuous fibers, but the local damage development pattern has changed. Materials containing SCF have higher bending stiffness and smaller deflections in the loading stage. However, once damage begins to initiate in the tensile region, cracks tend to localize and expand rapidly. This indicates that short carbon fibers increase the stiffness of the matrix and the local load transfer. For SKF materials, the samples maintain larger deflections and a more gradual damage process. Macroscopic damage observation results support the interpretation based on stress–strain and energy absorption results. The continuous fibers mainly determine the main deformation mode and failure trend. Carbon fiber-based composites exhibit higher stiffness but more concentrated and more sudden failure; while Kevlar fiber-based composites exhibit greater deformation and more gradual damage development, the short fiber-filled matrix further affects the local damage process by changing the stiffness of the matrix-dominated region and the resistance to crack propagation [30]. Therefore, the bending failure behavior of multi-scale hybrid materials should be understood as the combined result of continuous fibers controlling deformation and short fibers modifying the local damage evolution, rather than a simple linear superposition of the two reinforcing phases.

### 3.2. Interlaminar Peel Response and Representative Peeled-Surface Morphology

#### 3.2.1. Peel Load–Displacement Response

**Figure 6:**
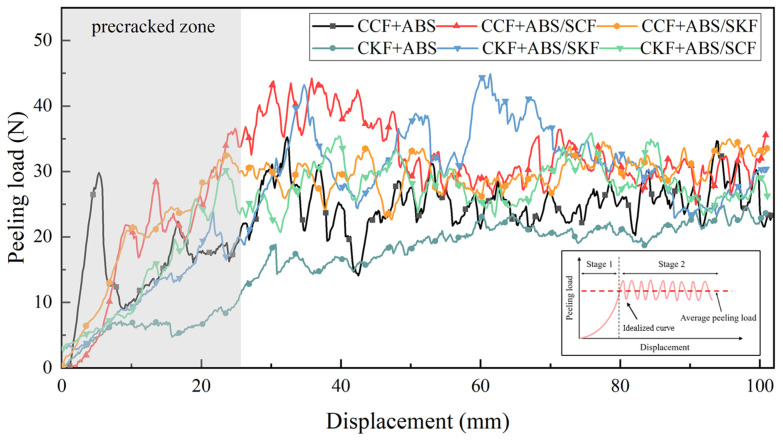
Representative peeling load–displacement curves.

The interlaminar delamination behavior of the printed composites was evaluated through the floating roller peel test. The typical delamination load–displacement curve of all samples is shown in Figure 6. All specimens exhibited typical staged delamination responses, including the initial crack initiation stage and the subsequent stable crack propagation stage. In the initial stage, the data were not used to calculate the average delamination load because the load response in this region was greatly affected by artificial pre-cracks, clamping conditions, and the initial relaxation degree of the flexible arm [33]. In the stable propagation stage, the delamination load fluctuated around an approximately constant average value, rather than remaining completely flat. This fluctuation indicates that the crack propagation in FDM-printed composites is not a uniform interface separation process, and the crack front repeatedly encounters changes in local resistance. When the crack extends to areas with higher local bonding strength or where there are fiber obstructions, the delamination load increases, while when local delamination, matrix tearing, or interfacial separation occurs, the load decreases [34]. Compared to the continuous fiber composite as the reference, the hybrid fiber composite typically showed a higher delamination load level in the stable propagation stage. This result indicates that the introduction of a matrix material filled with short fibers can effectively enhance the resistance of the composite to interlaminar crack propagation. Furthermore, by comparing the waveforms of the load, it can be observed that the curves of the hybrid fibers are more complex. This indicates that the influencing factors of interlayer separation in the hybrid fibers are more complex and require further detailed analysis.

The quantitative results of the peel tests are summarized in Table 5. The global one-way ANOVA revealed a highly significant variation among the investigated composite configurations (*p* < 0.001), justifying further pairwise comparisons via Tukey’s HSD test. For the continuous fiber reference composites, CCF + ABS has a higher stable peel resistance than CKF + ABS. The higher stiffness of carbon fibers enables them to provide stronger local resistance to the expansion of interlaminar cracks during the peel process. After introducing short fiber-filled matrix, all composites exhibited higher peel resistance than their corresponding continuous fiber reference materials. CCF + ABS/SCF has increased by approximately 30.46% compared to CCF + ABS, and CCF + ABS/SKF has an increase of approximately 18.66%. Compared to CKF + ABS, CKF + ABS/SKF has an increase of approximately 54.42%, and CKF + ABS/SCF has an increase of approximately 37.56%. These results confirm that introducing a short fiber-filled matrix can effectively enhance the interlaminar peel resistance of continuous fiber-reinforced composites. The post hoc Tukey’s HSD test confirmed that all multi-scale hybrid configurations exhibited significantly higher peel resistance than their corresponding continuous-fiber reference composites (*p* < 0.05). This result indicates that replacing the neat ABS matrix with a short-fiber-filled matrix improved the resistance to interlaminar crack propagation. In the comparison between homogeneous and heterogeneous hybrid composites, the homogeneous hybrid composites showed slightly higher mean peel resistance than the corresponding heterogeneous hybrid composites. However, this difference was not statistically significant (*p* > 0.05). The difference between homogeneous and heterogeneous hybridization should be interpreted as a numerical trend rather than a statistically confirmed advantage. From a materials perspective, the comparable peel resistance of the homogeneous and heterogeneous hybrid composites may be related to the different mechanical roles of carbon and Kevlar fibers. Carbon fibers generally contribute higher stiffness and local load-bearing capacity, whereas Kevlar fibers provide better deformation tolerance and energy-dissipation capacity [35]. Therefore, both homogeneous and heterogeneous hybrid configurations can contribute to interlaminar crack-growth resistance, although their differences in peel resistance were limited under the present testing conditions. This suggests that the short-fiber-filled matrix played a direct role in improving peel resistance, while the specific matching relationship between the continuous fiber and short fiber mainly affected the local failure morphology rather than producing a statistically significant difference in the measured peel resistance.

#### 3.2.2. Multi-Scale Peeled-Surface Morphology

**Figure 7:**
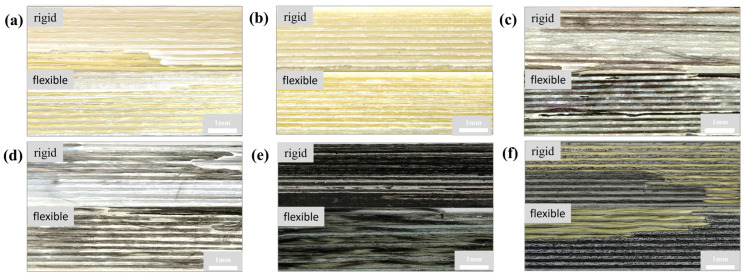
Representative peeled-surface morphologies of composites after floating roller peel tests: (**a**) CKF + ABS; (**b**) CKF + ABS/SKF; (**c**) CCF + ABS/SKF; (**d**) CCF + ABS; (**e**) CCF + ABS/SCF; and (**f**) CKF + ABS/SCF.

The peeled surfaces after the floating roller peel test were first observed by optical microscopy, and the results are shown in Figure 7. These optical images provide an overall view of the crack propagation path, printing layup traces, surface roughness, and visible interlayer separation features. Significant differences can be observed among different types of composites. For the benchmark continuous fiber-reinforced composite, the peeled surface showed relatively clear layup traces, indicating that the separation mainly occurs in the interlayer areas formed during printing. The peeled surface of the CKF + ABS composite is relatively smooth, while that of the CCF + ABS composite is more uneven, and there are more visible surface irregularities. After introducing short fiber-filled matrix, the peeled surface of the hybrid composite becomes rougher than the corresponding benchmark composite. More irregular peeling areas and local surface residues were observed on both the rigid and flexible sides, indicating that after the hybrid treatment, the crack propagation process became no longer uniform. Especially for the heterogeneous hybrid composite, there is a more significant difference in the peeled surfaces between the rigid and flexible sides, and the hybridization has changed the local separation behavior during the peeling process.

**Figure 8:**
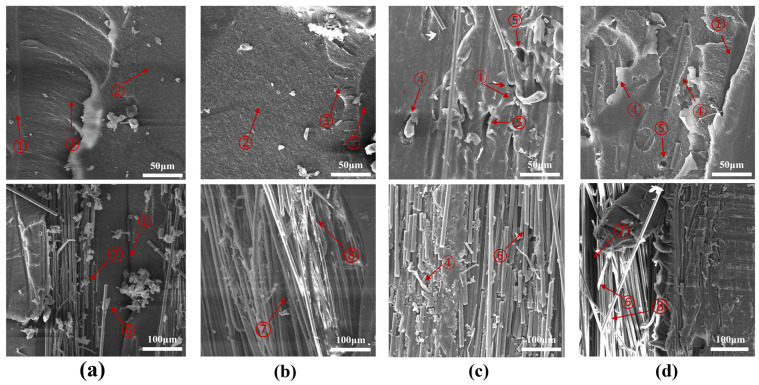
SEM images of the peeled surfaces of continuous fiber-reinforced composites and heterogeneous fiber-reinforced composites: (**a**) CCF + ABS; (**b**) CKF + ABS; (**c**) CCF + ABS/SKF; (**d**) CKF + ABS/SCF. The numbered labels ①–⑧ denote representative peeling-induced microstructural features.

To further analyze the peeling interface characteristics, SEM observations were focused on the two reference composites and the two heterogeneous hybrid configurations (Figure 8). As the peel performance between the homogeneous and heterogeneous composites showed no statistically significant differences, these four specimens were chosen as representative cases to examine the typical interlayer failure features.

As shown in Figure 8, clear deposition layer interfaces (label ①) were observed in all specimens, reflecting the layer-by-layer stacking characteristics of the FDM process. In CCF + ABS and CKF + ABS, the peeled surfaces contained relatively smooth matrix-dominated regions (label ②), with limited matrix residue and uneven peeling boundaries (label ③). Cracks mainly propagated along the fiber/matrix interface or the interlayer interface, indicating a relatively direct interfacial separation process. Local fiber/matrix separation and fiber pull-out (label ⑥) were also observed, suggesting that the interfacial adhesion was not fully sufficient in some regions. This phenomenon is closely related to the characteristics of FDM-printed continuous fiber-reinforced thermoplastic composites, where limited consolidation pressure, possible voids (label ⑦), insufficient fiber wetting, and weak interlayer diffusion can restrict interfacial bonding and interrupt effective stress transfer [6,36]. After introducing the short-fiber-filled matrices, the hybrid composites exhibited rougher and more irregular peeled surfaces. Compared with the reference composites, the smooth matrix-dominated regions decreased, while matrix fragments, local tearing features, discontinuous crack boundaries, exposed fibers, and fiber pull-out traces became more evident. In the short Kevlar fiber-filled matrix, deformed fibers and fiber pull-out traces (labels ④and ⑤) were observed, indicating that SKFs altered the crack propagation path in the matrix during peeling. In the short carbon fiber-filled matrix, most SCFs remained embedded in the ABS matrix, with only a few local fiber pull-out traces (label ⑤), while irregular interfacial failure features were still observed. This suggests that the rigid SCFs caused local stress concentration around the fiber/matrix interface, resulting in a more complex interfacial failure morphology.

The observed interfacial separation also helps explain the macroscopic mechanical results. On the one hand, local interfacial separation indicates that part of the applied load could not be transferred through a fully bonded interface, which may limit the reinforcing efficiency of the fibers in flexural loading. This helps explain why the improvement in flexural modulus and flexural strength was not simply proportional to the introduction of short fibers. On the other hand, during the peel test, matrix tearing, fiber pull-out, interfacial debonding, crack-path deflection, and bridging-like features (label ⑧) near the continuous fibers can increase energy dissipation during interfacial separation. Therefore, the hybrid composites still exhibited higher interlaminar peel resistance than the corresponding continuous-fiber reference composites. The improved interlaminar peel resistance can thus be attributed to the combined effects of matrix stiffening, crack-path deflection, matrix tearing, fiber pull-out, and additional energy dissipation during interfacial separation [37].

### 3.3. Quantitative Hybrid Effect and Design Implications

To assess the deviation of the measured flexural properties of the hybrid composites from the corresponding RoM reference values, a RoM-based comparison was conducted. For the two bulk flexural performance indicators, namely flexural strength and flexural modulus, the corresponding hybrid effect *HE* values were calculated. The calculated *HE* values are summarized in Table 6.

All four hybrid configurations showed positive *HE* values in terms of flexural strength and flexural modulus. This result suggests that the measured flexural properties of the hybrid composites were higher than the corresponding RoM reference values, indicating positive deviations associated with the introduction of short-fiber-filled matrices. However, the strength of the hybrid effect varies significantly among different fiber combinations and performance indicators. In terms of flexural performance, the CCF + ABS/SCF configuration exhibited the largest positive deviation, with an *HE* value of +24.86% for flexural strength and +24.88% for flexural modulus. This result is consistent with its highest measured flexural modulus and strength data, indicating that the homogeneous carbon/carbon fiber hybrid configuration performs most outstandingly in enhancing bending stiffness and load-bearing capacity. Among all hybrid configurations, CCF + ABS/SKF exhibited the lowest hybrid effect, with *HE* values of only +6.62% for flexural strength and +5.32% for flexural modulus. This result is consistent with the previous flexural analysis, as the limited stiffness contribution of SKFs and the rapid failure of the CCF-based structure restricted the activation of SKF-related deformation coordination during loading. In contrast, the composites containing SKF showed relatively lower *HE* values for flexural performance, suggesting that SKF’s effectiveness in enhancing the bending-related hybrid effect is less than that of SCF.

The RoM analysis indicates that the optimal hybrid structure design depends on the targeted performance indicators. Among them, the CCF + ABS/SCF structure performed most outstandingly in bending stiffness and strength. While the RoM analysis focuses on bulk flexural properties, the experimental results in Section 3.2.1 demonstrate that all hybrid configurations yield an improved interlaminar peel resistance. Therefore, the CKF + ABS/SCF structure emerges as a highly balanced configuration, achieving outstanding theoretical flexural synergy while maintaining improved interlaminar delamination resistance. These research results provide comparative evidence for evaluating the hybrid effect of different multi-scale carbon fiber/Kevlar fiber hybrid structures.

## 4. Conclusions

This paper introduces multi-scale carbon fiber/Kevlar fiber hybrid reinforcement into FDM-printed ABS-based composites. Through the combination of CCF or CKF with short carbon fiber or short Kevlar fiber fillers within the ABS matrix, different hybrid configurations of composites are constructed. Their flexural behavior, interlaminar peel resistance, fracture surface morphology, and hybrid effects based on the Rule of Mixtures are systematically investigated. The main conclusions are summarized as follows:

The bending response is mainly controlled by the type of continuous fibers. CCF-based composites show higher stiffness and strength but more brittle failure. In contrast, CKF-based composites show lower stiffness and strength but better deformation capacity and more gradual damage. The introduction of short fiber fillers in the matrix can further improve the flexural performance of both CCF-based and CKF-based composites. In addition, the effect depends on fiber matching. Among them, CKF + ABS/SCF, although it has a lower flexural modulus and strength than CCF + ABS/SCF, SCF effectively compensates for the insufficient stiffness of the CKF-based system, while CKF retains better deformation coordination ability and gradual damage characteristics, thus forming a more balanced response between bearing capacity and deformation limit.

All hybrid composites show higher interlaminar peel resistance than the corresponding single continuous-fiber composites. The introduction of short fiber fillers in the matrix can strengthen the matrix-dominated interlayer area, improving the material’s ability to resist interlayer crack propagation. The peeled surface morphology further indicates that after introducing short fiber fillers in the matrix, the peeled surface becomes rougher and more irregular, and more obvious matrix damage, fiber pull-out, and crack path deflection features appear, indicating that the improved peel resistance was associated with a more tortuous interlaminar failure process involving matrix damage, fiber pull-out, and crack-path deflection. Overall, both homogeneous and heterogeneous hybrid configurations successfully yield a robust and statistically comparable enhancement in peel resistance, indicating that the multi-scale strategy effectively improves interlaminar peel resistance regardless of the continuous/short fiber combinations.

The RoM-based comparison showed that all hybrid systems exhibited positive deviations from the corresponding RoM reference values in terms of flexural properties. These deviations indicate that the measured flexural properties were higher than those obtained from the selected RoM reference, and may be related to the combined effects of continuous fibers, short-fiber-filled matrix, and printed interlayer interfaces. CCF + ABS/SCF has the largest positive deviation in flexural strength and flexural modulus, indicating that a homogeneous carbon/carbon hybrid is more suitable for high stiffness and high strength design. Concurrently, the CKF + ABS/SCF configuration achieves an optimal balance, showing positive deviations in flexural properties while simultaneously improving interlaminar peel resistance.

Overall, multi-scale carbon fiber/Kevlar fiber hybrid reinforcement is an effective strategy for controlling the flexural performance and interlayer performance of FDM-printed ABS-based composites. The mechanical response of the material is influenced by multiple factors, such as the type of continuous fibers, the filling of short fibers in the matrix, the continuity of the matrix, the resistance to crack propagation, and the fiber matching relationship. These results provide guidance for the design of printed hybrid composites with improved flexural performance and interlaminar peel resistance. In future work, the influence of fiber content and fiber arrangement will be further investigated to better understand the relationship between structural design and mechanical performance. In addition, more comprehensive mechanical tests, including tensile and fracture, will be carried out to further evaluate the failure behavior of printed hybrid composites.

## Figures and Tables

**Table 1:** Material parameters of ABS-based matrix filaments and continuous fibers used in this study. (**a**) ABS-based matrix filaments [24,25], (**b**) Continuous fibers [26,27].

(a)						
Material	Reinforcement Type	Filament Diameter (mm)	Density (g/cm^3^)	Fiber Length (μm)	Fiber Diameter (μm)	Short Fiber Content (wt.%)
ABS	None	1.75	1.03	-	-	-
ABS/SCF	Short carbon fiber	1.75	1.05	251	5.8 ± 0.5	2.48
ABS/SKF	Short Kevlar fiber	1.75	1.04	215	12 ± 0.6	4.42
**(b)**						
**Material**		**Reinforcement Type**	**Density (g/cm^3^)**		**Linear Density (tex)**	
1 K carbon fiber		Continuous carbon fiber	1.77		67	
600D Kevlar fiber		Continuous Kevlar fiber	1.44		66.7	

**Table 2:** Composite configurations and classification of the investigated specimens.

Category	Specimen	Short-Fiber-Filled Matrix	Continuous Fiber
Baseline continuous-fiber composite	CCF + ABS	—	CCF
Baseline continuous-fiber composite	CKF + ABS	—	CKF
Homogeneous hybrid composite	CCF + ABS/SCF	SCF	CCF
Homogeneous hybrid composite	CKF + ABS/SKF	SKF	CKF
Heterogeneous hybrid composite	CCF + ABS/SKF	SKF	CCF
Heterogeneous hybrid composite	CKF + ABS/SCF	SCF	CKF

**Table 3:** Volume-fraction parameters used for the RoM-based hybrid effect calculation.

Composites	Continuous-Fiber Volume Fraction	Matrix Volume Fraction
CCF-based composites	10.30%	89.70%
CKF-based composites	12.31%	87.69%

**Table 4:** The flexural modulus and flexural strength of composite.

Specimen	Flexural Modulus (MPa)	Flexural Strength (MPa)
CCF + ABS	7852.36 ± 494.70	149.34 ± 8.66
CKF + ABS	5733.75 ± 424.30	135.83 ± 9.24
CCF + ABS/SCF	10,532.51 ± 579.29	190.06 ± 10.07
CKF + ABS/SKF	7573.45 ± 613.45	157.96 ± 12.00
CKF + ABS/SCF	7540.29 ± 505.20	172.23 ± 10.68
CCF + ABS/SKF	8435.90 ± 725.49	153.32 ± 12.27

**Table 5:** Peel properties of all composite configurations.

Specimen	Average Peel Load (N)	Peel Strength (N/m)
CCF + ABS	25.25 ± 1.27 ^b^	2003.38 ± 193.35 ^b^
CKF + ABS	20.52 ± 1.52 ^b^	1627.95 ± 178.44 ^b^
CCF + ABS/SCF	32.93 ± 2.34 ^a^	2613.60 ± 219.37 ^a^
CKF + ABS/SKF	31.68 ± 3.57 ^a^	2513.96 ± 247.56 ^a^
CCF + ABS/SKF	29.95 ± 2.85 ^a^	2377.34 ± 209.98 ^a^
CKF + ABS/SCF	28.53 ± 2.75 ^a^	2239.42 ± 278.15 ^a^

Note: Data are presented as mean ± standard deviation (*n* = 5). Means within a column followed by different superscript letters (a, b) indicate statistically significant differences (*p* < 0.05), whereas shared superscript letters denote that the differences are not statistically significant (*p* > 0.05), determined by one-way ANOVA followed by Tukey’s HSD post hoc test.

**Table 6:** Hybrid effect coefficients of different hybrid configurations.

Hybrid Configuration	*HE* of Flexural Strength (%)	*HE* of Flexural Modulus (%)
CCF + ABS/SCF	+24.86	+24.88
CCF + ABS/SKF	+5.32	+6.62
CKF + ABS/SCF	+24.17	+19.40
CKF + ABS/SKF	+19.60	+30.72

## Data Availability

The original contributions presented in this study are included in the article. Further inquiries can be directed to the corresponding author.
